# The hopes of West African refugees during resettlement in northern Sweden: a 6-year prospective qualitative study of pathways and agency thoughts

**DOI:** 10.1186/1752-1505-6-1

**Published:** 2012-01-24

**Authors:** Tanvir M Anjum, Cecilia Nordqvist, Toomas Timpka

**Affiliations:** 1 Centre for Refugee Medicine, Östergötland County Council, Norrköping, Sweden; 2 Department of Medical and Health Sciences, Linköping University, Linköping, Sweden

**Keywords:** Refugee mental health, West African refugees, Psychology of hope, Qualitative methods

## Abstract

**Background:**

Little is known about how positive phenomena can support resettlement of refugees in a new country. The aim of this study was to examine the hopeful thinking in a group of West African quota refugees at arrival and after 6 years in Sweden and compare these thoughts to the views of resettlement support professionals.

**Method:**

The primary study population comprised 56 adult refugees and 13 resettlement professionals. Qualitative data were collected from the refugees by questionnaires on arrival and 6 years later. Data were collected from the resettlement professionals by interview about 3 years after arrival of the refugees. Snyder's cognitive model of hope was used to inform the comparative data analyses.

**Results:**

Hopes regarding education were in focus for the refugees shortly after arrival, but thoughts on family reunion were central later in the resettlement process. During the later stages of the resettlement process, the unresponsiveness of the support organization to the family reunion problem became as issue for the refugees. The professionals reported a complex mix of "silent agency thoughts" underlying the local resettlement process as a contributing reason for this unresponsiveness.

**Conclusion:**

Hopes regarding education and family reunion were central in the resettlement of West African refugees in Sweden. These thoughts were not systematically followed up by the support organization; possibly the resources for refugees were not fully released. More studies are needed to further investigate the motivational factors underpinning host community support of refugees' hopes and plans.

## Background

With an estimated 214 million people on the move internationally and approximately 750 million people migrating within their own country, population mobility is among the leading policy issues of the 21st century. If internal and international migrants comprised a nation, it would be the third most populous country in the world, just after China and India [1]. The West African nations Liberia and Sierra Leone produced a considerable number of refugees as a result of a brutal civil war in 1991-2002 [2,3]. Rival factions were responsible for the deaths of more than 300,000 civilians (250,000 in Liberia; 50,000 in Sierra Leone), including an estimated 50,000 children. Mass terrorization of entire villages and towns was routine. Common practices included summary execution, systematic rape of women, use of children as soldiers, and destruction of property (Additional file 1[4]). In 2000, Guinea was host to the largest concentration of refugees on the continent of Africa, hosting nearly 480,000 individuals from these countries. Even though several studies have reported from programs implemented in refugee camps [5,6], less is known about resettlement and integration of West African refugee groups in countries outside Africa [7,8]. Previous research has shown that refugee resettlement is influenced by aspects in contrasting areas of human existence. Exposure to severely traumatic events in their home country, and the medical and psychological sequelae of these exposures are known to have a critical influence on the possibilities for resettlement in a new country [9]. However, the post-migration environment, in particular the social circumstances, also influences this process. For example, in a group of Iraqi refugees, a low level of social support in exile was a stronger predictor of depressive morbidity than trauma factors [10]. Similarly, in a refugee group representative of small unit operations of war with low access to early medical care, the degree of physical disability was not a salient factor for social integration 2 years after arrival in the new country [11]. Research has thus suggested that economic difficulties, discrimination and language difficulties are among the most important resettlement challenges for refugees [12,13].

Said [14] summarizes exile as an "unhealable rift between a human being and a native place, between the self and its true home". As such, exile precipitates a "condition of terminal loss" caused by a "discontinuous state of being". Thus, the influences on resettlement among refugees range from cultural differences and obstacles to reconciling personal hopes to ongoing conflicts in the country of origin jeopardizing the safety of family and friends [15]. This study focuses on a specific set of these influences, namely positive psychological phenomena associated with a successful resettlement. It sets out to explore how hopes are related to the integration of West African refugees in Swedish society. The specific aim was to examine the hopeful thinking of a group of quota refugees from Liberia and Sierra Leone on arrival and after 6 years in Sweden and compare these thoughts to the views of resettlement support professionals. For the study, we define the term *hope *as goal thoughts in Snyder's cognitive model of hope [16]; the term hope is associated with both "pathways thoughts" and "agency thoughts" (motives). Resettlement and integration are processes that occur between refugees and host communities over an extended period of time, in which the refugees have to integrate their personal hopes with the meaning structures of two different cultures [17]. Such a process requires long-term, reliable, and responsive communication and understandings between the receiving society and refugees [18] across different social, economic and political arenas [19].

According to Snyder et al. [16], hope is "a positive motivational state that is based on an interactively derived sense of successful agency (goal-directed energy), and pathways (planning to meet goals)". Hopeful thinking thus involves both the perceived capacity to envision workable routes and the goal-directed energy needed to explore these routes. Pathways and agency thoughts iterate, and together they lead to attachment of value to desired goal outcomes. If a particular goal is important enough to enable prolonged cognitive processing, the individual starts the event sequence by initiating agency and pathways thoughts. Goals are defined as the targets of these mental action sequences, reflecting imagined end points that the person desires [20]. These goals must be attainable, yet contain some uncertainty. Goals with certain attainment do not necessitate hope. In order to reach their goals, people must be able to produce viable routes. Such "pathways thinking" reflects evaluations of own proficiency in producing plausible routes to goals. Although pathways thought involves the perceived capability of generating at least one effective route to a desired end point, thoughts of multiple pathways often become available when encountering impediments to goals. Here, high-hope people see themselves as being able to come up with alternate routes, whereas low-hope people do not [21]. Agency is the motivational component in Snyder's model. It reflects thoughts about the power to proceed along imagined pathways to goals. As such, agency taps affirmative thoughts that can initiate and sustain movement along the pathways that are invented to desired goals. Agentic thinking is particularly relevant when initial routes are blocked and there is a need to channel motivation to appropriate alternate pathways [22].

## Methods

The study used a longitudinal design with data collection on arrival in Sweden and 6 years later. Qualitative data were collected by postal questionnaires from refugees and interviews with representatives from the resettlement support organizations. The data were analyzed using summative content analyses. The study design was approved by an institutional review panel at the Swedish Migration Board.

### Study setting

In spring 2001, 113 natives of Sierra Leone and Liberia were selected by the Swedish Immigration Board in cooperation with UNHCR (United Nations High Commission for Refugees) as quota refugees at the UNHCR office in Guinea. In May-June 2001, these refugees arrived in two small municipalities (populations of 3400 and 8700) in northern Sweden. When the West African group arrived, both these municipalities had regularly received quota refugees from other countries for about 10 years. Their agreement with the national government was to supply resettlement services to the refugees against an annual reimbursement of 15,000 euros per adult and 9000 euros per child. The municipalities were each reimbursed 46,000 euros per year for the administrative overheads without any restriction on the number of refugees. After arrival, all refugees participated in a 2-week full-time introduction program [23]. Social welfare officers and psychologists conducted an interview with each of the refugees. The interview included discussions on social background, earlier experiences and present psychosocial health. After the introduction program, an individual short-term action plan was established in cooperation with the employment agency. Central to the program was an ambitious Swedish language training plan, including work experience in the labour market arranged by the employment agency. The idea was to have an early focus on measures for gaining employment. The refugees were also offered treatment for psychosocial trauma. Local mentors were appointed to the refugee groups to facilitate contact between the refugees and local government and administration.

### Primary study group

The first author met the group of West African quota refugees (*n *= 113) in their community when they arrived in Sweden in 2001 and informed them about the study. All approached were positive about participating in the study and had the option to withdraw without giving any reason. All 56 individuals 16 years of age and older in the group were approached and gave their written consent to participate.

The sample professional representatives of the resettlement support organizations (*n *= 13) was chosen to represent the different professions involved with the study group and both municipalities, including municipal administrative managers, refugee coordinators, language teachers, counselors, and mentors.

### Data collection

Data were collected from the refugees using a resettlement questionnaire in English, the official language of Sierra Leone and Liberia, constructed on the basis of previous studies of refugee resettlement carried out in Scandinavian settings [11-15]. The baseline questionnaire distributed to the refugees at the time of arrival included questions asking for sociodemographic data, and self-reports of hopes and future plans.

After 6 years living in their communities, an identical follow-up questionnaire was sent to the study group by mail. One new open-ended question was added at the follow-up on their views about choosing Sweden as the migration country.

The data from professional representatives of the resettlement support organizations were collected by personal semi-structured interviews. The interview questionnaire was constructed on the basis of previous studies of refugee resettlement carried out in Scandinavian settings [11-15]. The questions targeted the professionals' views about the chances of integration of the study group and what could potentially hinder this process. The interviews were performed once, about 3 years after arrival, that is, when the professionals had finished the initial and most intense phase of the work with the group of refugees.

### Data analyses

Descriptive quantitative data from the baseline questionnaires were analyzed using statistical methods. The analyses were performed using the SPSS software package (version 14). The data from the open-ended question in the refugee questionnaire and from the interviews with the professionals were transferred to documents and analyzed using summative content analysis [24,25] in seven steps: (1) the texts were read by the first author independently to obtain a general view; (2) the data were separated with regard to source (refugee, professional) and time for collection (arrival, follow-up); (3) all authors met to discuss and devise a preliminary coding scheme informed by Snyder's cognitive theory of hope; (4) coded statements were sorted into categories and subcategories were identified; (5) latent content analyses were then established successively as agreement was reached after multiple discussions, focusing on the similarities and differences by comparing the codes and then looking for the same underlying ideas regarding goal, pathways, and agency thoughts to be categorized together; (6) the final codes were established by the authors and then sorted into categories and subcategories within each of the main themes; (7) the themes were compared for similarities and differences.

## Results

Sociodemographic data for the primary study group (*n *= 56) at the time of arrival in Sweden is given in Table 1. According to the local population records 6 years after arrival, one of the participants had died and another had moved out of the country. Of the remaining 54 refugees, 28 (52%) responded to the second resettlement questionnaire. The mean age at arrival of respondents was 29.2 years compared with 26.1 years for the dropouts. Men were slightly over-represented among dropouts (57.1%) compared with the primary population (46.4%). Almost everyone in the primary population had experienced traumatic incidents in the home country; 91% had been involuntarily separated from family, 87% had witnessed torture/killings, and 98% had experienced someone important to them being killed. Overall, there were few differences between respondents and dropouts at 6 years after arrival.

**Table 1:** Sociodemographic data for the primary study group (*n *= 56) at the time of arrival divided into respondents at the 6-year follow-up (*n *= 28) and dropouts (*n *= 28).

	Respondents		Dropouts		Total	
	
	*n*	Percent	*n*	Percent	*n*	Percent
Sex						
Male	10	35.7	16	57.1	26	46.4
Female	18	64.2	12	42.8	30	53.5
Religion						
Christian	16	57.1	21	75.0	37	66.0
Muslim	10	35.7	6	21.4	16	28.5
No reply	2	7.1	1	3.5	3	5.3
Marital status						
Married	13	46.4	10	35.7	23	41.0
Unmarried	11	39.2	16	57.1	27	48.2
Divorced	0	0	0	0	0	0
Widow	2	7.1	0	0	2	3.5
No reply	2	7.1	2	7.1	4	7.1
Education						
University	10	35.7	8	28.5	18	32.1
Secondary	11	39.2	13	46.4	24	42.8
Primary	3	10.7	7	25.0	10	17.8
No education	4	14.2	0	0	4	7.1
Self-reported health good	11	39.2	12	42.8	23	41.0
Regular social interaction outside family	4	14.2	7	25.0	11	19.6
Involuntarily separated from family	26	92.8	25	89.2	51	91.0
Witnessed torture/killings	26	92.8	23	82.1	49	87.5
Anyone important killed in home country	28	100	27	96.4	55	98.2

### Refugee's accounts of hopes as factors in the resettlement process

At arrival, getting an education and establishing a professional career were the main goal thoughts expressed by the respondents. Following are comments from respondents at arrival. Gaining language skills was a common pathways thought expressed by the refugees.

I am hoping to study the language, get a job, and settle down in Sweden....

Several of the respondents expressed concrete plans and pathways thoughts about how to achieve educational and professional goals, although acknowledging that the journey could be long and tiresome. For instance, several young women had qualified to attend nursing college, and some of them had already become registered nurses. The dream of getting a good higher education and to be able to serve Sweden, their host country, was also common.

My hope is to become a business woman in the future. I want to learn how to speak Swedish language, and then start a business.

Agency thoughts were mentioned less often at arrival. Some refugees said they wanted to get an education and thereafter be able to help their home country. In particular, being able to help the children was an important motive in this case.

To further my education, get a job. That one day I will give a helping hand to poor children in my country.

Regarding hopes after 6 years, the main focus for goal thoughts was the family, and in particular the fact that the refugees had been separated from many of their family members. The forced exile disrupted family and cultural systems, and separated many refugees from their original ethnic community. Almost all the participants in our study reported missing family members after their arrival in Sweden. Many of them did not even know where their family members were. Several of the group members reported that they succeeded in contacting family members and some of them had got visas on a kinship basis to reunite with their families in Sweden. Yet, at the 6-year follow-up, some refugees reported that their family members, although they had got in touch with them, were still in Africa. The refugee's hopes and future plans regarding family reunion at follow-up are summed up in the following extracts. In contrast to the situation at arrival, agency thoughts, usually associated with perceived family responsibilities, were commonly expressed at the 6-year follow-up.

... [I hope] that my late sister's children will be able to join me and my family here in Sweden. I am responsible for their schooling and feeding back in Liberia.

... Last but not least, help my younger brothers and sister to join me in Sweden, because I am their only hope alive.

Finding pathways to family reunion was expressed to be both particularly important and difficult in the West African refugee context. There were shown to be substantial cultural differences from mainstream northern European culture in terms of identity and belonging. In particular, the definition of "family" differs in Sweden and West Africa. These refugees considered family members on a broader perspective; brothers and sisters more than 20 years of age, their children, and older parents are also family members for which all responsibilities are to be shared.

My hope is for my brother and his family to come and join me here. My sister tried hard for him. We even went for an interview, but the migration rejected him. They told us the man is old enough to take care of himself and his family. [But it] is our responsibility now to help him.

In Sweden, in comparison, *kärnfamilj *(core family) means only husband and wife and their children less than 20 years of age. This discrepancy between views caused problems for this refugee group. Feelings of guilt were common as they considered themselves responsible for taking care of family still living under difficult conditions in Africa.

### Resettlement support professional's accounts of hopes as factors in the resettlement process

The resettlement professionals' hopes for the West African refugee group early in the resettlement process, in harmony with the refugees themselves, were strongly focused on education and introduction to the Swedish labour market. The open social structure in both resettlement communities was described as giving the refugees easy opportunities for social contact with local people and within their own group. Accordingly, communication-related pathways thoughts were highlighted, as expressed by the opinion of this professional:

... the young age of most of the refugees, their religion and church activities, and their ability to speak English makes it easier [for them] to communicate with Swedish people.

The professionals believed that this refugee group had the potential to become better integrated than most previous refugee groups with which they had experience, for example, those from the Middle East. However, although in the time period immediately after the refugees' arrival the focus for pathways thoughts were shared between most categories of resettlement professionals, this was not the case regarding agency thoughts. There was a suspicion among the professionals that the communities were hosting refugees "out of self-interest" rather than for humanitarian motives. Several of them said that they believed that the financial reimbursement from the national government was the main incentive for the municipality boards' continuing willingness to accept quota refugees. They pointed out that policy makers did not meet with the refugees, and were therefore not aware of many of the realities on the ground. Moreover, several of the professionals described alternative motives for inviting the refugees to resettle in the actual communities. For instance, they explained that the local parishes may have played an operational role in the arrival of the West African group to the communities. The professionals mentioned that they had noticed that some of the refugees already had host families before they came to Sweden, and that these families were active in the local church. In addition, there were structural associations between the local resettlement organization, the local church, and the foreign aid organization in Guinea that selected quota refugees for Sweden. The respondents suspected that there had been a bias towards choosing Christian refugees, basing this argument on the fact that two-thirds of the refugees arriving in Sweden were Christians, compared with less than one-third of the population in Sierra Leone. They also pointed out that "the previously half-empty church now was filled with refugees". The professionals in the resettlement support organization were positive about the future possibilities for the West African refugees in Sweden, despite the fact that they believed that there was a complex mix of "silent host motives" underlying the local resettlement process responsible for the lack of an integrated plan for sustainable resettlement.

Although the pathways thoughts were seemingly shared early on during the resettlement, during the later stages there were few concrete pathways and motivational factors that could underpin implementation of integration plans. Most importantly, there was no systematic arrangement for support of family reunions or to help the refugees attend more than elementary education programs. The resettlement services were instead described as having been provided reactively to the refugee group, "on an event basis'. Most of the services were short-term and practical by nature, for example, providing housing and access to schools. Symptomatically, the professionals who had had most personal contact with the refugees were the Swedish teachers. The teachers described that they had had to "carry more load" than they were able (and willing) to. They reported that they often had to take on other roles than those covered by their professional tasks. Many of the refugees came to them with other than language-related issues, for example, mental health problems such as anxiety and restlessness. Many refugees were worried about their families, which made them unable to focus on learning Swedish. The teachers expressed that they found that the refugees "got money but not the emotional support they needed". They also believed that it was unreasonable to ask the refugees to attend 750 hours of Swedish language classes in only two semesters. They felt that they received little support from other professionals in the resettlement support organization when they pointed out these problems. However, the teachers also pointed out that the lack of sustainable resettlement support was not only because of insufficiencies in the host communities. For instance, to promote physical activities among the refugees, they received financial support to visit the gymnasium and swimming pool. Even though the entrance fees were heavily subsidized, the refugees chose not to use this opportunity and instead sent money to relatives who had remained in Africa.

## Discussion

This study set out to examine the hopeful thinking of a group of West African quota refugees from Liberia and Sierra Leone at arrival and after 6 years in Sweden and compare these thoughts to the views of resettlement support professionals. While hopes regarding education were in focus for the refugees at arrival, thoughts on family reunion were central later on in the resettlement process. It was found that unresponsiveness of the support organization to the family reunion problem compromised refugee hopes during the later stages of the resettlement. Progress among the refugees had thus materialized despite the lack of a long-term program in response to the refugees' hopes and aspirations. Based on the fact that the study group consisted of quota refugees, such a program could have been expected to exist. Instead, central to the resettlement support organization were ambitious thoughts about pathways towards the short-term integration of the refugees in the local host communities [23]. The professionals in the support organization suspected that a complex mix of "silent agency thoughts" underlying the resettlement process in the local community contributed to the lack of a long-term program. These silent host motives, which were not mentioned in official planning documents, included that the municipality government regarded the reimbursement for the refugees from the national government as a means to balance budget deficits, and that the local church needed the refugees as members to keep their activities running. In other words, even though the host communities and the refugees had found common motivational grounds for social and economic co-development, the support actions provided by the support program were not coordinated for the dominant long-term needs and wishes of the refugees. As a substitute, the resettlement organization was guided by short-term obligations determined by the agreement with the national government and the host communities' own needs. In comparison, describing settlement of Sierra Leonean and Liberian refugees in Guinea [26], a refugee assistance program supported self-settlement, leading to the refugees developing partial self-sufficiency. The settlement pattern of the refugees largely determined the degree of self-sufficiency they could reach. However, also in that setting, the details of the refugees' situation were not sufficiently acknowledged by the aid agencies. As found in this study also, recognizing these details is a precondition for assistance that complements the refugees own coping mechanisms. Snyder and Feldman [27] have described several advantages of having hope-engendering authorities, i.e. government systems that are "fair" and responsive. If refugees perceive that the directives of the host authorities do not unfairly impede their goal-directed life activities, such as striving for family reunion, they should be less likely to experience frustration. Moreover, such hope-engendering authorities decrease the probability that a large segment of refugees will simply stop trying and not fully release their resources. The observations made in this study suggest that unambiguous directives regarding family reunion and absence of silent motives are fundamental requirements of such hope-engendering resettlement support authorities.

Many of the refugees in this study were quite young on arrival, having no professional education. Other immigrants or refugees arriving later in life often have a profession or professional education that sometimes is not recognized. Such experiences can lead to feelings of humiliation and have negative effects on hopeful thinking. In a study of Ethiopian refugees in the United Kingdom [28], a major problem for many of the participants was finding an occupation that was comparable with their education and skills. Because of their young age, the present study group had more opportunities to focus their hopes on getting an education and building a career. Education and employment are sources of contact with out-groups and provide scope for networking and building social capital into the wider community [29]. Evaluations have shown that intensive Swedish language courses, such as those provided to the present study group, provide students with a better knowledge of Swedish than immigrants participating in normal Swedish courses [30]. Most of the study group thus had sufficient language skills to be able to transform their goals into concrete pathways to higher studies. This result is in agreement with a study [28] in which young refugees from Ethiopia reported that they adapted more quickly, learning the language and accents better than older people. Even though the refugees had managed to some extent to harmonize hopes with tangible future plans in the area of education and working life, this was not the case in the area of family reunion. Swedish authorities consider every refugee case as an independent case and not as kinship. "Good resettlement" [31] of newly arrived young people requires not only a service system that is accessible and has multiple entry points, but where young people have ample opportunity to develop bonding and bridging relationships. Loss of significant loved ones in the migration process and social isolation in exile cannot be ignored. Trauma and prolonged family separation have been observed to have a negative effect on emotions and identity [32]. Many of the refugees in our study mentioned that they were still struggling to get their family members into Sweden. Restoration of family and community bonds seems to be a more decisive motivational factor for many refugee groups than economic benefits. In a study on Somali refugees in Norway, it was found that the United Kingdom seems to be the main destination for Somalis leaving Norway, despite less generous welfare benefits [33].

According to the resettlement support professionals, church membership and religious activities among the refugees were associated with increased hope in the resettlement and integration process. It is interesting that other researchers mention that Somalis were not particularly religious in Somalia or during their first period in Norway, but after a while became extremely religious and rigid in their views, and used religion to justify a negative view of Norwegian culture [33]. The religion a person is born and raised with may affect the person, and religious belief may give the person hope throughout his/her life outside their native country. Interpretation of the same religion can be different from person to society to country [34]. In their study, Papadopoulos et al. [28] mentioned that the refugees' religious beliefs and practice gave them hope, guidance, continuity, familiarity and spiritual support during their struggle to adapt to British culture. In her study on women immigrants from Ghana in Canada, Donker [35] found that the bond of religion and/or cultural unity gave women the common purpose they needed to adjust. Cultural associations helped group cohesion, and the churches further strengthened group solidarity. The role of religion in relation to hope and resettlement is yet to be explored and deeper investigations of these issues are warranted.

This study has several limitations that need to be taken into consideration when interpreting the results. The 50% dropout rate is notable but not unexpected. A dropout rate of 40-60% is not uncommon in longitudinal studies of the present type [36]. Our dropout analysis did not reveal any exceptional differences between responders and non-responders. The study may therefore still contribute to the question of how these refugees from West Africa rebuild their lives in exile. However, generalizations to other refugees groups should be made with care. Moreover, there may be differences between various West African refugee populations with regard to resettlement conditions, for example, between refugees from Sierra Leone and Liberia. Such differences were not explored in the present study. Neither did we investigate the qualities included in the integration that the refugees expected to be part of in Sweden.

## Conclusion

It is argued [37] that resettlement of refugees depends on a range of factors including the policies of the country of asylum and the experiences and attitudes of individuals to exile. We found that West African refugees' hopes regarding education and family reunion were central during resettlement in Sweden. We also identified factors in the receiving society that promoted and inhibited these hopes. More studies are needed to further investigate the motivational factors underpinning the host community support of refugees' hopes and plans. The two municipalities in Sweden were willing to host the resettlement of this quota refugee group, even though the motives may not all have been humanitarian.

## Competing interests

The authors declare that they have no competing interests.

## Authors' contributions

TA participated in the design of the study, carried out the data collection, participated in the analyses and drafted the manuscript. CN participated in the design of the study, carried out the analyses. TT conceived of the study, and participated in its design and coordination and helped to draft the manuscript. All authors read and approved the final manuscript.

## Supplementary Material

Additional file 1 **Conflict background**. Brief summary of the conflict background and development in Sierra Leone and Liberia 1989-2002. Click here for file
