# Supplementary material for: The hopes of West African refugees during resettlement in northern Sweden: a 6-year prospective qualitative study of pathways and agency thoughts
Source: Confl Health. 2012 Jan 24;6:1. doi: 10.1186/1752-1505-6-1 (PMC3284866; doi:10.1186/1752-1505-6-1)
Supplement: Additional file 1 — Conflict background. Brief summary of the conflict background and development in Sierra Leone and Liberia 1989-2002. [file 1752-1505-6-1-S1.DOC]

# The hopes of West African refugees during resettlement in northern Sweden: a 6-year prospective qualitative study of pathways and agency thoughts

# Additional file 1

# Conflict background

In Liberia in 1989, the National Patriotic Front of Liberia, led by Charles Taylor, launched an uprising against the Samuel Doe government. In 1990, a Nigerian-led West African regional force (ECOMOG) was dispatched in an effort to establish peace, but later that year, rebels from a splinter group murdered the president, and Liberia descended into a civil war. For the next 5 years, there were several tentative ceasefires and disarmament efforts, interrupted by re-escalations of violence. Between 1989 and 1996, hundreds of thousands of Liberians fled across the border to seek refuge in Guinea. By August 1996, a measure of stability appeared to have been re-established, and large numbers of these refugees were repatriated. In July 1997, Charles Taylor was elected president. War erupted again in 1999 when anti-Taylor forces invaded Liberia; Taylor’s forces launched a massive offensive against these rebels the following year. In 2002, more than 50,000 Liberians and Sierra Leoneans fled the escalating violence to seek refuge in neighbouring countries. The results of initial needs assessments in Guinea indicated that these experiences of war had left profound psychological scars. A large proportion of the refugees were suffering from depression, lethargy, hopelessness, anxiety, and post-traumatic stress. Many were unable to make meaningful contributions to their families and communities. Most found it difficult to imagine rebuilding their lives [4]. In June 2003, Taylor was indicted by the United Nations–backed Special Court for Sierra Leone for war crimes committed on Sierra Leonean territory; 2 months later, as the rebels closed in on Monrovia, Liberia, Taylor was forced into exile, and peace was restored. At the end of March 2006, Charles Taylor was extradited from Nigeria and brought to Freetown, Sierra Leone, to stand trial in the Special Court.

The war in Liberia spread across the border into Sierra Leone in 1991, when the Revolutionary United Front, led by Foday Sankoh and supported by Charles Taylor, launched an uprising against the democratically elected president. This precipitated a long period of instability and violence, including three military coups and a failed peace agreement over the following 7 years. The situation appeared to have been stabilized in 1998, when with the assistance of ECOMOG, the rebels were driven out of Freetown, and the president returned from exile in Guinea. In January 1999, however, the Revolutionary United Front invaded Freetown, killing 5000 people. The United Nations intervened, a ceasefire was established, a peace agreement between the government and the rebels was signed, and United Nations troops arrived to enforce this agreement. Nevertheless, for the next 2 years, the situation remained unstable. In May 2001, disarmament of the rebel forces finally began, and in January of the following year, the war was declared over.

War atrocities described by refugees from Liberia and Sierra Leone include intentional hacking off of limbs, carving the initials of rebel factions into victims’ skin, slaughtering pregnant women to bet on the gender of the unborn child, and use of young girls as human sacrifices. Children and teenagers were forced to watch the torture, rape, and brutal murders of their parents and siblings. In many cases, family members—including children—were forced to rape, murder, and mutilate each other. In regions where attacks occurred, large portions of the population had to hide in the dense and inhospitable forest for long periods without food or water, and had to walk for many miles to seek refuge, often falling into ambushes along the way. Large numbers were extorted and humiliated when attempting to cross national borders into safety [3].
